# Medical Fees to Fellow Practitioners

**Published:** 1918-05-18

**Authors:** 


### The Hospital

The Workers' Newspaper of Administrative Medicine and Institutional Life, Administration, National Insurance and Health.

No. 1667, Vol. LXIV. SATURDAY, MAY 18, 1918. PRICE ONE PENNY

### MEDICAL FEES TO FELLOW PRACTITIONERS.

The unwritten rules and customs which regulate the relations of medical practitioners to one another are not always easily understood by the general public, but they certainly ought to be understood by members of the profession. That this is not always so must be concluded from a case recently heard in the King's Bench Division of the High Court of Justice, when, in answer to a claim for the payment of fees for professional services rendered to a medical practitioner's wife, the defendant urged that such services must be considered gratuitous, "in accordance with the custom and etiquette of the profession." As a matter of fact, the defendant won his case, but he relied upon answers other than the one just quoted, and we do not gather that the decision involved any judicial sanction for the plea with which we are here concerned. What may be the legal force of "custom" in such a matter it is not for us to say, but we should certainly be surprised were the doctrine advanced by the defendant to be authoritatively approved as a proposition endorsed by the law of the land. It is, however, the case that it was advanced by the defence, and, indeed, it was stated in the broadest possible terms. Not only the practitioner's wife, it seems, was entitled to gratuitous treatment, but also his "family," and this was defined as a legal right established by the "custom and etiquette of the profession." For our own part, we venture to doubt this interpretation, and, while allowing that the practice exists in greater or less degree, we regard it wholly as an expression of good will, and not as a bond of obligation. Still further, we believe that in many instances the custom might well be suspended, and this with advantage to all the persons concerned. For this conclusion we are prepared to give our reasons.

First, on the question of fact, we recognise that it is the custom for a member of the profession to abstain from charging fees for professional services rendered to a colleague, and not infrequently the generosity is extended to a colleague's wife and even to his family. At times, too, the "family" is given a wide interpretation. But until legal authority can be quoted against us we shall believe that all such proceedings are voluntary, not compulsory. Further, we are quite certain that the practice is not universal, and that in departing from it there is nothing which can be called a breach of "etiquette." If one likes to give and another to receive there is nothing more to be said, but neither on one side nor on the other is there anything unworthy in refusing such an arrangement. The matter is one for private settlement between the persons interested, and the practice accepted by some has no binding effect on the methods preferred by others. In a word, while custom necessarily exercises some degree of force, departure from it involves no reflection either on the one who pays or on the one who receives payment. It is a fair and honest principle that service shall receive its reward, and exceptions to this principle require justification. There is thus a *prima facie* case against the custom here discussed, and this is our first reason for urging that its application might well be restricted within narrower limits than is at present the case.

In the second place we would say that not only is the labourer worthy of his hire but, speaking generally, it is under this condition that the most whole-hearted work is done. Not for a moment do we doubt that the practitioner serving his colleague gives his best service without any thought of fee. Indeed, his conscientious desire in this direction may lead him to an amount of attention beyond what he would give to a lay patient. But it is inevitable that at times the burden should become a heavy one, and that it may prove a hindrance to work carrying monetary recognition. Indeed, in particular instances claims of this order may be so numerous that they can only be accepted at a heavy sacrifice. Hence we would completely alter the incidence of the "custom," and would make it the rule that professional services rendered to colleagues should be met by the usual fees, unless good reasons exist for making an exception in any individual instance.

If so much may be said on behalf of the donor, still more may be urged from the side of the recipient. It is surely a sound social principle that no man should be compelled to accept an obligation against his will. Grant this, and it becomes obvious that the individual, whether a medical practitioner or another, who prefers to pay for professional services rendered to him ought to be free to do so. Neither the "custom" of the profession nor the obstinacy of the individual adviser has a right to be heard against his independent choice. Assume that he is able to pay and that he wishes to pay, his voice, it seems to us, ought to command the situation. There is, apart from the question of sentiment, at least one important practical advantage inherent in such a position. It is that further help may be freely asked. If this has to be asked as an additional obligation hesitation may well arise, and most certainly does arise. But, alter the circumstances, and the practitioner-patient has the same freedom as that enjoyed by the layman. At present he is often hindered in this respect, and at the best is driven to hunt for some form of present which may or may not be of service to the one who receives it. Thus it seems to us to be in the interest of both parties that the "custom" should be that professional services should be met by professional fees. To say this, however, is not to prescribe the relations between personal friends or between colleagues closely associated in professional work; still less is it to suggest that the generous traditions of the profession should be sacrificed where gratuitous and unselfish help is needed. In each of these directions there may well be both readiness to give and readiness to accept. But we do say that the present "custom," when applied apart from the conditions just mentioned, is apt to be unjust both to the adviser and to the patient. From a neighbourly and fraternal habit it has become extended so as to act not infrequently as a penalty to the one and a source of embarrassment to the other, and thus we feel compelled to advocate its relaxation.

